# Type 2 Diabetic Retinopathy Screening in a General Practice: A Five-Year Retrospective Analysis

**DOI:** 10.7759/cureus.11713

**Published:** 2020-11-26

**Authors:** Anthony J Chung, My Nhi Dang

**Affiliations:** 1 Medicine and Surgery, Queen Elizabeth Hospital Woolwich, London, GBR; 2 General Practice, Imperial College London, London, GBR

**Keywords:** types 2 diabetes, diabetic retinopathy, diabetes mellitus, screening, primary care

## Abstract

Aim

To assess current standards of diabetic retinopathy screening in primary care against the National Institute of Clinical Excellence (NICE) guidelines for type 2 diabetes mellitus. Moreover, to determine whether individuals with diabetes were screened for diabetic retinopathy no later than three months from referral to the local eye screening service and no later than one year from their last retinal screen.

Materials and methods

A single-center, retrospective audit was undertaken at a small general practice. Data was collected from the health records of individuals placed on the type 2 diabetes register from 01/01/2013 to 01/01/2018. Individuals who were diagnosed with diabetes whilst registered at a different practice, who had pre-diabetic retinal screening or were referred onto a different screening pathway were excluded. A total of 50 records were audited and data collection involved demographics, dates individuals were placed on the diabetes register and dates of attendance and non-attendance to screening.

Results

16.0% of individuals with type 2 diabetes underwent retinal screening which adhered to the NICE guidelines. Of the cohort which did not adhere, 59.5% experienced an interval greater than three months between diagnosis and first retinal screening and 64.3% experienced a screening interval greater than one year.

Conclusions

Diabetic retinopathy screening of individuals must be improved to meet the NICE standards. Interventions should be implemented to increase the awareness within general practitioners and practice nurses to ensure all people with diabetes receive their first retinal screen within the first three months of diagnosis with regular annual screening thereafter.

## Introduction

Diabetic retinopathy, a common complication of both type 1 and type 2 diabetes mellitus, is the single largest cause of blindness before old age. However, it takes several years for diabetic retinopathy to progress to a stage where sight is threatened. Diabetic Eye Screening, a national screening program introduced in 2003, was initiated with the aims of reducing the risk of sight loss through early detection and treatment, where necessary, of sight-threatening retinopathy. This was achieved through examination of the retina via mydriatic digital photography and is offered to all people with diabetes aged 12 years and over. By 2008, the screening program achieved population coverage across the entirety of England. In 2015-16, the annual uptake of the program was 82.8% [1].

In 2012, the Royal College of Ophthalmologists (RCOphth) authored Diabetic Retinopathy Guidelines, providing valuable clinical guidance for the management of diabetic eye disease [2]. The RCOphth guidelines make particular reference to the National Institute of Clinical Excellence (NICE) guidelines and supports the use of population-based digital photography screening program for diabetic retinopathy in the United Kingdom. The current standards outlined for adults with type 2 diabetes [3] state that on diagnosis, general practitioners should immediately refer adults with type 2 diabetes to the local eye screening service; perform screening as soon as possible and no later than three months from referral; arrange repeat structured eye screening annually.

This retrospective audit was undertaken with the aim of determining whether current diabetic retinopathy screening in patients registered to one general practice adhered with the NICE guidelines. In particular, this audit assessed whether individuals with type 2 diabetes had undergone screening for diabetic retinopathy no more than three months from referral to the local eye screening service. Furthermore, we investigated whether people with diabetes had undergone retinal screening no later than a year from their most previous screen. Compliance to these objectives would be determined by obtaining retinal screening dates from health records.

This article was previously presented as an oral presentation at the Advanced Ophthalmologic Practice 2020 Annual Scientific Meeting on January 11, 2020.

## Materials and methods

A single-center audit was undertaken at a small general practice serving approximately 6000 patients. Data was collected retrospectively and extracted using Vision healthcare software and Docman electronic document management software. All cases read-coded as “Type 2 diabetes mellitus. Placed on register” over the period of five years (1st January 2013 - 1st January 2018) were audited. The audit was registered and approved in line with general practice processes.

**Figure 1:**
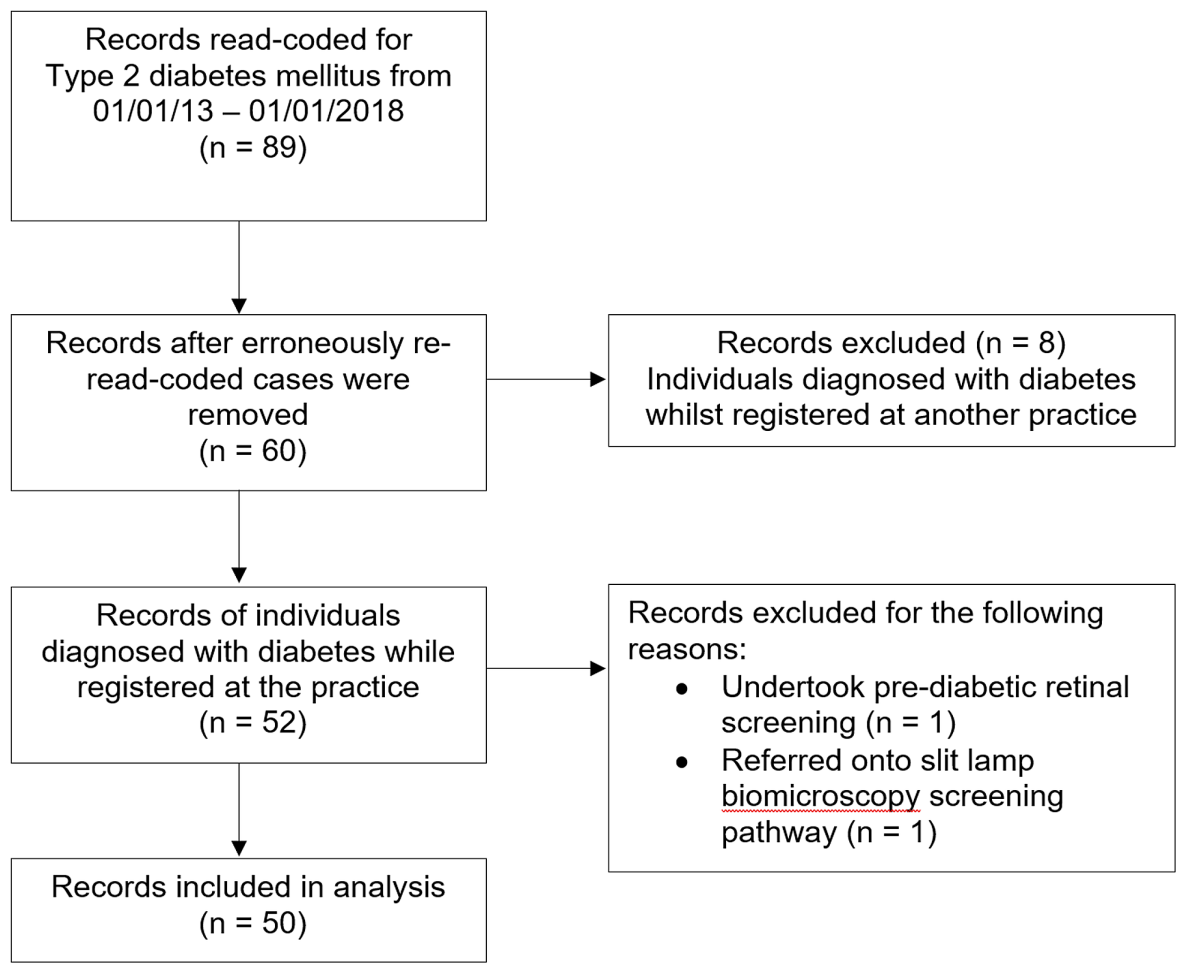
Flow chart of selection of patient records for inclusion in audit analysis.

A total of 89 people were added to the the type 2 diabetes register during the data collection period (Figure 1). After examining patient documentation, 29 individuals had been diagnosed with diabetes prior to the data collection period despite being read-coded, and thus were excluded. Additionally, only individuals registered to the practice prior to their diagnosis of diabetes were included. Individuals who had been diagnosed with diabetes in other practices prior to registering with the audited practice were excluded. Furthermore, one person was excluded due to attendance at pre-diabetic retinal screening and another person was excluded due to referral onto a slit lamp biomicroscopy screening pathway. After exclusion, 50 cases were included in this study. The audit collected data on the following domains: age and gender, dates individuals were placed on the diabetes register, dates of attendance and non-attendance to diabetic retinopathy screening and postponements to screening appointments.

## Results

Of the 60 people diagnosed with type 2 diabetes in this audit period, 50 fulfilled the relevant audit criteria and were thus included in the analysis. As seen in Table 1, the overall mean age for people with type 2 diabetes at diagnosis was 59.5 years, with a range from 33 to 83 included. Additionally, there were a greater number of males (n = 34) compared to females (n = 16) in this audit. 

**Table 1:** Clinical and demographic information for all patients included in analysis.

Variable	Patients (n = 50)
Demographics	
Age, years	59.5 (33-83)
Female, n (%)	16 (68.3)
Male, n (%)	34 (55.4)

**Figure 2:**
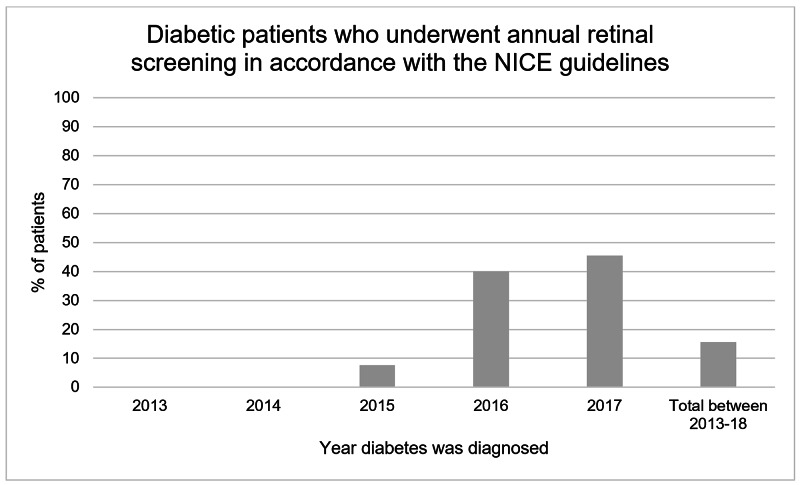
Percentage of diabetic patients diagnosed between 2013-2018 who underwent annual retinal screening in accordance with the NICE guidelines.

In the audit period, 16.0% of people with type 2 diabetes were screened for diabetic retinopathy in line with NICE guidelines. No persons diagnosed with diabetes in 2013 and 2014 had retinal screening which fully met the criteria defined by the NICE (Figure 2). From 2014 onwards, the percentage of individuals with diabetes who underwent satisfactory screening increased with each consecutive year. There was a large increase in the percentage of individuals fulfilling the NICE criteria between those diagnosed in 2015 (8.3%) and 2016 (40.0%). The increasing trend peaks with individuals diagnosed with diabetes in 2017 (45.5%).

**Figure 3:**
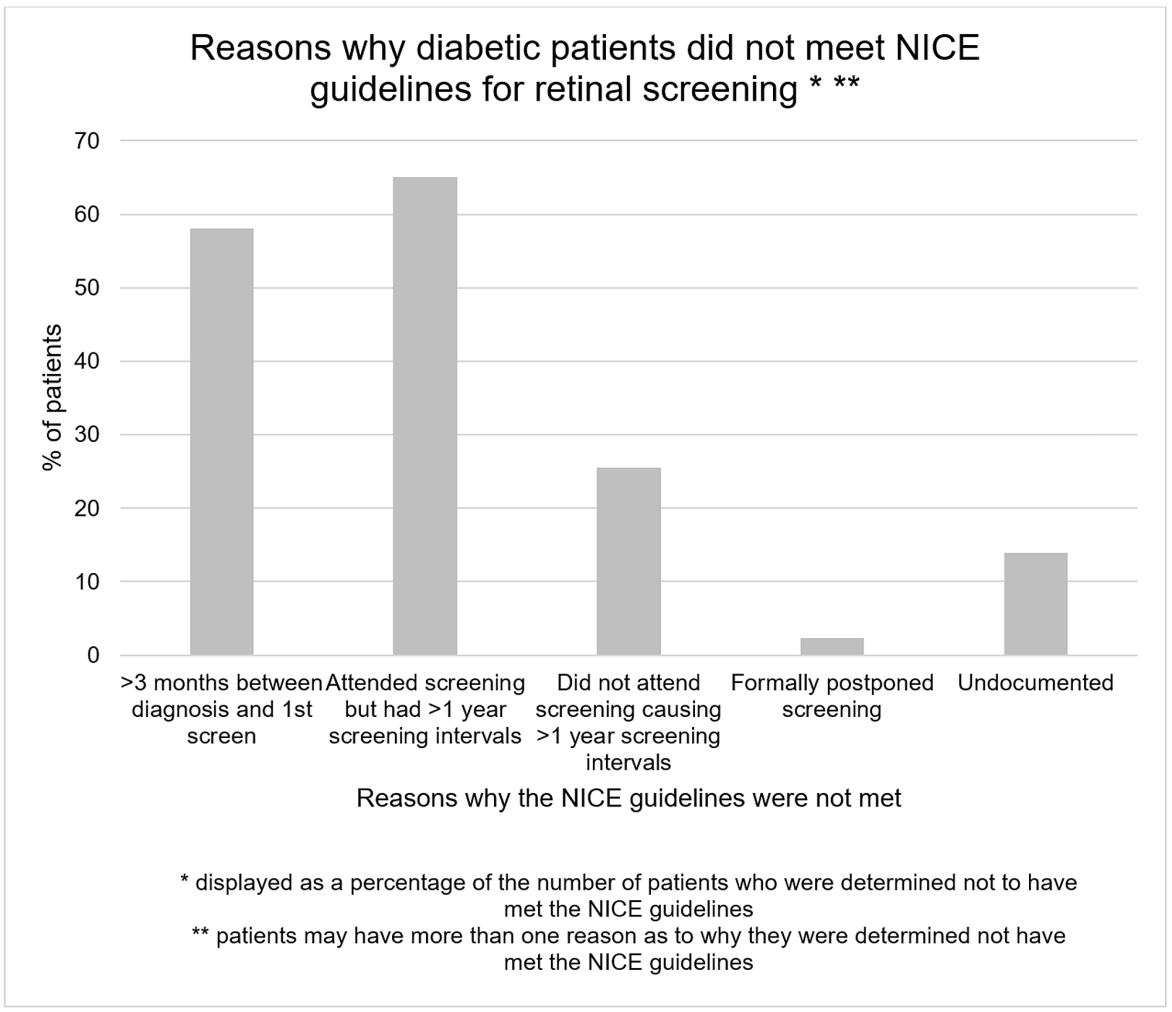
Reasons why diabetic patients diagnosed between 2013-2018 were determined not to have met the NICE retinal screening guidelines.

Of the 42 individuals determined not to have met the NICE guidelines, 59.5% had a time period of greater than three months between their diagnosis and their first appointment, 64.3% attended screening but had screening intervals which were greater than a year and 26.2% did not attend their scheduled screening appointments (Figure 3). One individual (2.4%) had formally postponed their screening appointment while six patients’ records (14.3%) contained missing documentation regarding whether they were screened or not.

**Figure 4:**
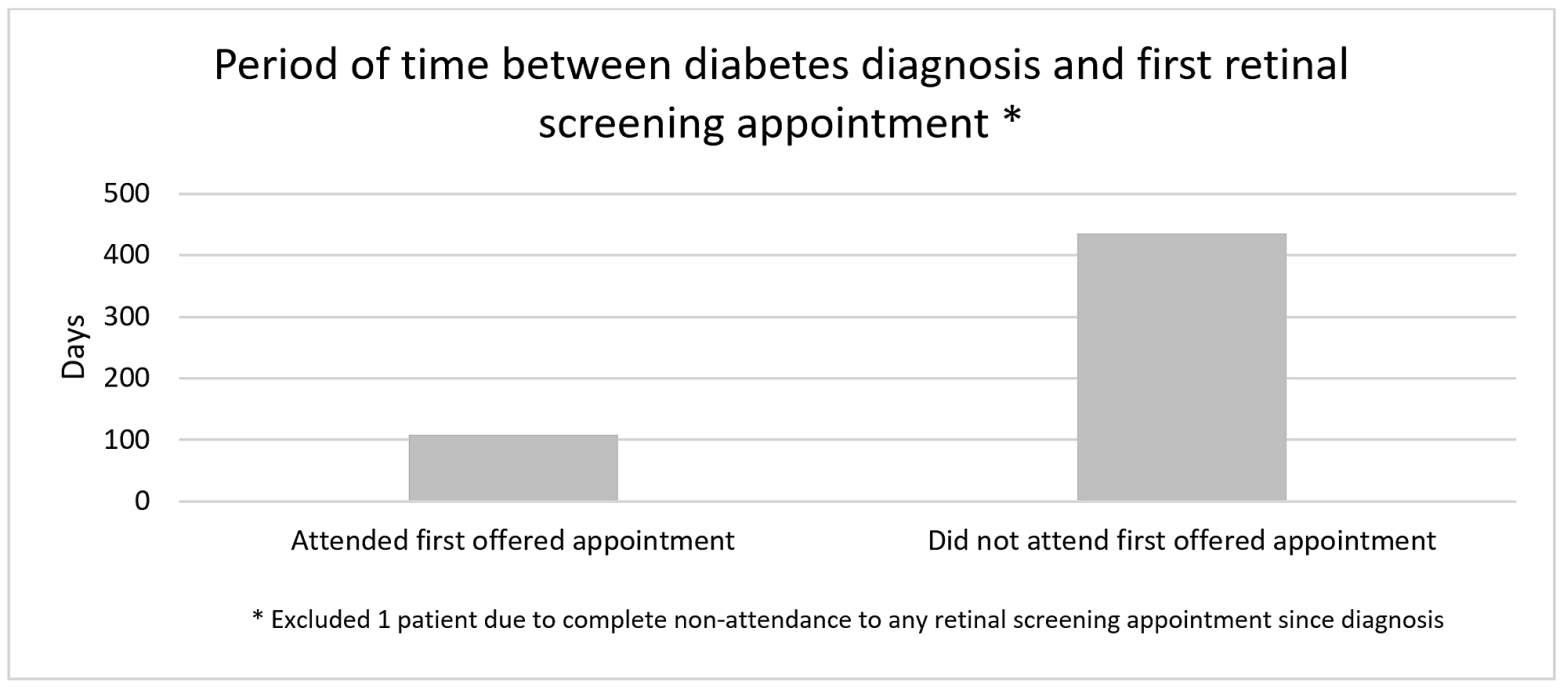
Average period of time between diabetes diagnosis and first retinal screening appointment.

Of the individuals who attended their first offered retinal screening appointment, the mean period of time between diagnosis and their initial screening appointment was 108.7 days (Figure 4). In comparison, individuals who did not attend their first offered retinal screening appointment underwent their first retinal screen an average of 435.2 days following their date of diagnosis.

In summary, 16.0% of individuals included in this audit had diabetic retinal screening which completely fulfilled the NICE criteria, this includes attending the diabetic screening program within three months from their referral and screening within one year from their last retinal screening appointment. Of the individuals who did not meet the NICE guidelines, 59.5% were due to the interval between the diagnosis date and first screening being greater than three months, and 64.3% were due to screening intervals of greater than one year in length. Moreover, there is a 326.5 day difference in the time taken from diagnosis to first retinal screening appointment between individuals who attend their first appointment and those who do not attend their first appointment.

## Discussion

This audit was undertaken with the aim of determining whether current diabetic retinopathy screening in people with type 2 diabetes registered to one general practice in London adhered with the NICE guidelines. Furthermore, by identifying areas of improvement, implementing an intervention and re-auditing at a later stage, this audit provides the groundwork to evaluate whether progression has been made in aligning current retinal screening practices with those imposed by the NICE as part of the United Kingdom's National Health Service (NHS) retinopathy screening program.

Main findings

This audit revealed a positive trend between the year of diagnosis and the percentage of people with type 2 diabetes who underwent retinal screening in accordance with the NICE guidelines. As seen in Figure 2, no people diagnosed with diabetes in 2013 and 2014 underwent retinal screening which fully complied with the NICE guidelines. However, with each consecutive year of diagnosis from 2014, the percentage of individuals compliant with the NICE guidelines progressively increases. This may be explained through several factors. Firstly, reduced awareness of the NICE guidelines and lack of diabetic screening pathway training in healthcare professionals in the past may have contributed to the low compliance rate. Additionally, changes in patient attitudes towards attending screening programs may account for these results. Patient attitudes may be influenced by greater patient education on the detrimental effects of diabetic retinopathy [4], social media usage, NHS promotional materials and increased recommendations by healthcare providers [5]. Despite the improvement in compliance rate, people diagnosed with diabetes in 2017 still fell short of the standards outlined by the NICE guidelines, with only 45.5% successfully undergoing retinal screening.

Our in-depth examination of the reasons underlying why diabetic retinopathy screening guidelines were not being achieved identified two main causes (Figure 3). The primary reason was that, despite patient attendance, screening intervals were greater than the NICE-recommended one-year period (64.3%). Secondly, the time period between diagnosis and the first screening appointment was found to be greater than the NICE-recommended maximum of three months (59.5%). Both findings may be explained through a multitude of factors, including administrative errors, long waiting lists, delayed referrals and rescheduling of appointments.

Furthermore, 26.2% of people with diabetes did not attend their scheduled screening appointments. It was beyond the scope of our study to identify the reasons as to why people did not attend their screening appointments, however, this topic has been explored in detail in the literature [6-8]. In particular, Strutton et al. performed a quantitative analysis to identify explanations for why individuals registered to one South London diabetic eye screening program had never attended a screening appointment [8]. The study categorized factors given to non-attendance into patient-level factors and system-level factors. Patient-level factors were factors determined to some extent by the patient while system-level factors were those determined by the healthcare provider. The study established that reasons for non-attendance included patients having other commitments, patient anxiety and miscommunication regarding the patient’s address and clinical condition. The results of the study by Strutton et al. are useful in providing insight into our study as our cohort are of similar demographic and geographic population [8].

It is important to highlight that 14.3% of people with diabetes had incomplete documentation regarding diabetic screening appointments. This included lack of documentation concerning dates of screening appointments and patient attendance. Whilst missing data is especially common in any retrospective study, greater attention could be made in future to minimize the amount of missing data for re-audits. This can be achieved through the implementation of a proforma which would aid healthcare professionals in submitting completed documentation.

Our study emphasizes the importance of following up individuals with diabetes who do not attend their first screening appointment. As seen in Figure 4, individuals who did not attend their first offered screening appointment are seen on average 326.5 days later compared to individuals who did attend their first offered appointment. It appears that non-attendance to the first offered screening appointment is a strong indicator that the individual will not have their first screen until greater than a year’s length in time has passed.

Furthermore, our audit has revealed that despite attendance to the first offered screening appointment, the average number of days between diagnosis and the aforementioned is 108.7 days. This is slightly greater than the maximum three month interval as outlined by the NICE suggesting that system-level factors are the predominant cause of the failure to adhere to the NICE criteria.

Strengths and limitations

There are many strengths associated with this study. This audit incorporated a large number of people with diabetes during its phase one period, thus allowing it to stand in good stead for future comparisons among other phases. Additionally, by collecting data over a five year period, this study had a vast data set and was able to identify reliable trends. Finally, the retrospective nature of this study ensures that the data collected was not influenced by any changes in behavior or patient care by healthcare providers.

Whilst our study was able to provide valuable insights into the different factors as to why diabetic retinopathy screening in primary care patients are not currently in line with NICE guidelines, there are some limitations to note. Our study utilized the date individuals were placed on the diabetes register in substitute of the date of referral to the local eye screening service. This likely resulted in an overestimation of people determined to have not met the NICE criteria, as the date of referral is typically a few days after the date individuals were placed on the diabetes register. 

Moreover, the shortcomings associated with the single-center study design of this audit should be considered. The lack of external validity in single-center studies means that the results of this study may not be representative of individuals registered to other practices. Differences in patient demographics, varying attitudes and socioeconomic disparities make it difficult to generalize our results to the patient population. This combined with the different priorities of healthcare providers across the country introduces further challenges against the applicability of our results. Also, it would have been useful to collect data on travel distance to the nearest screening center as this is a major factor influencing the elderly population of diabetic individuals whom are at a greater risk of developing diabetic complications.

## Conclusions

Overall, this audit explored the key factors that influence whether individuals with type 2 diabetes receive retinopathy screening in accordance with the NICE guidelines. This includes long periods of time between diagnosis and screening, screening intervals of greater than a year, non-attendance and lack of documentation. Thus, several interventions are recommended to improve adherence with diabetic retinopathy screening guidelines. Interventions can be classified into those aimed at healthcare professionals and those aimed at individuals with diabetes.

Healthcare professionals in general practice need increased awareness of the diabetic retinopathy NICE guidelines. This may be facilitated through annual training sessions, as well as including this as a mandatory topic of discussion in general practice meetings. Targeted reminder notifications on Vision software to prompt action from healthcare professionals may be helpful in practice. Additionally, implementation of a proforma tailored towards people with diabetes will encourage complete documentation from healthcare professionals. Regular audits are essential to assess compliance with the NICE standards. We also recommend incorporation of regular follow ups for individuals with diabetes who do not attend their first offered screening appointment as this cohort are particularly at risk of delayed retinopathy screening . This could be through an escalation in contact methods including text messages, letters and phone calls. 
